# A phase I clinical study of autologous dendritic cell therapy in patients with relapsed or refractory multiple myeloma

**DOI:** 10.18632/oncotarget.14582

**Published:** 2017-01-10

**Authors:** Sung-Hoon Jung, Hyun-Ju Lee, Youn-Kyung Lee, Deok-Hwan Yang, Hyeoung-Joon Kim, Joon Haeng Rhee, Frank Emmrich, Je-Jung Lee

**Affiliations:** ^1^ Department of Hematology-Oncology, Chonnam National University Hwasun Hospital, Hwasun, Jeollanamdo, Republic of Korea; ^2^ Research Center for Cancer Immunotherapy, Chonnam National University Hwasun Hospital, Hwasun, Jeollanamdo, Republic of Korea; ^3^ Research Institute, Vaxcell-Bio Therapeutics, Hwasun, Jeollanamdo, Republic of Korea; ^4^ Department of Microbiology, Chonnam National University Medical School, Gwangju, Republic of Korea; ^5^ Fraunhofer Institute for Cell Therapy and Immunology, Leipzig, Germany

**Keywords:** dendritic cell, VAX-DC/MM, immunotherapy, multiple myeloma

## Abstract

Cellular immunotherapy is emerging as a potential immunotherapeutic modality in multiple myeloma (MM). We have developed potent immunotherapeutic agent (VAX-DC/MM) generated by dendritic cells (DCs) loaded with autologous myeloma cells irradiated with ultraviolet B. In this study, we evaluated the safety and efficacy of VAX-DC/MM in patients with relapsed or refractory MM. This trial enrolled relapsed or refractory MM patients who had received both thalidomide- and bortezomib-based therapies. Patients received the intradermal VAX-DC/MM injection every week for 4 weeks. Patients were treated with 5 × 10^6^ or 10 × 10^6^ cells, with nine patients treated at a higher dose. The median time from diagnosis to VAX-DC/MM therapy was 56.6 months (range, 28.5–130.5). Patients had received a median of five prior treatments, and 75% had received autologous stem cell transplantation. VAX-DC therapy was well-tolerated, and the most frequent adverse events were local reactions at the injection site and infusion-related reactions. In seven of nine patients who received 10×10^6^ cells, an immunological response (77.8%) was observed by interferon-gamma ELISPOT assay or a mixed lymphocyte reaction assay for T-cell proliferation. The clinical benefit rate was 66.7% including one (11.1%) with minor response and five (55.6%) with stable disease; three (33.3%) patients showed disease progression. In conclusion, VAX-DC/MM therapy was well-tolerated, and had disease-stabilizing activity in heavily pretreated MM cases. Further studies are needed to increase the efficacy of VAX-DC/MM in patients with MM.

## INTRODUCTION

Multiple myeloma (MM) is a clonal B-cell neoplasm characterized by aberrant expansion of malignant plasma cells within bone marrow [1]. The prognosis for patients with MM has improved substantially with high-dose chemotherapy/autologous stem cell transplantation and the development of new, effective agents [2, 3]. However, most of patients with MM ultimately relapse, with resistance to prior treatment. Thus, treatments for patients with relapsed or refractory MM are needed to improve survival outcomes; this is an important focus of research.

Cellular immunotherapy using dendritic cell (DCs) has been considered an alternative treatment modality to conventional chemotherapy for patients with relapsed or refractory MM [4]. The efficacy of cellular immunotherapy using DCs in MM is based on the observation that allogeneic stem cell transplantation is curative for a subset of patients with MM because of the graft-versus-myeloma effect [5]. In addition, this effect is supported by the disease response following donor lymphocyte infusions [6]. However, allogeneic stem cell transplantation does not have immune activity specific to myeloma cell and may be associated with significant morbidity and mortality. DCs-based immunotherapy may selectively target myeloma cells while minimizing toxicity to normal cells. Rosenblatt J *et al*. [7] reported that vaccination with DCs/myeloma fusions was safe and resulted in an antitumor immune response. In addition, disease stabilization was seen in a majority of patients. For this reason, several basic or clinical studies have been performed to generate potent DCs for immunotherapy in MM.

Recently, we have developed a potent immunotherapeutic agent (VAX-DC/MM) that consists of DCs loaded with autologous myeloma cells irradiated with ultraviolet B (UVB). In this study, we evaluated the safety and efficacy of VAX-DC/MM in patients with relapsed or refractory MM.

## RESULTS

### Patient characteristics

In total, 16 patients were screened between December 2013 and January 2015, and 15 met the eligibility criteria. Two patients were excluded because the cell yield of mononuclear cells obtained by leukapheresis was not sufficient to generate VAX-DC/MM. One patient was excluded due to an inadequate amount of myeloma cells obtained by bone marrow aspiration. Thus, 12 patients underwent VAX-DC/MM therapy. Baseline clinical characteristics of all patients are presented in Table 1. The median age was 62.5 years (range, 47-75) and 5 (41.6%) patients were over 65 years old. According to the International Staging System (ISS), five (41.7%) patients had ISS III, five (41.7%) had ISS II, and two (16.7%) had ISS I at initial diagnosis. The median time from diagnosis to VAX-DC/MM therapy was 56.6 months (range, 28.5-130.5). Patients had received a median of five (range, 2-8) prior treatment regimens. All patients received the thalidomide, cyclophosphamide, and dexamethasone (CTD) regimen and bortezomib, cyclophosphamide, and dexamethasone (VCD) regimen as initial or salvage therapy. Six patients (50%) received the bortezomib retreatment (VCD regimen) before the VAX-DC/MM therapy. In addition, nine (75%) patients had received high-dose chemotherapy and autologous stem cell transplantation and two underwent tandem autologous stem cell transplantation. When enrolled, four patients (25%) were double refractory to both a bortezomib and thalidomide, 8 patients (66.6%) were refractory to thalidomide, and 4 (25%) were refractory to bortezomib. One showed the refractoriness to lenalidomide, cyclophosphamide, and dexamethasone therapy.

**Table 1:** Baseline clinical characteristics of patients (*n* = 12)

Variables	
Median age, year (range)	62.5 (47-75)
Gender, *n* (%) Male Female	5 (41.7%) 7 (58.3%)
Immunoglobulin (Ig) type, *n* (%) IgG IgA	8 (66.7%) 4 (33.3%)
International staging system, *n* (%) I II III	2 (16.7%) 5 (41.7%) 5 (41.7%)
Previous treatments Median no., (range) Thalidomide, *n* (%) Bortezomib, *n* (%) Lenalidomide, *n* (%)	5 (2-8) 12 (100%) 12 (100%) 1 (8.3%)
Performance of ASCT, *n* (%)	9 (75.0%)
Median time to VAX-DC/MM therapy	56.6 (28.5-130.5) months

Abbreviations: *n*, number; ASCT, autologous stem cell transplantation

### Characteristics of VAX-DC/MM

In this study, the phenotypes of monocyte-derived immature DCs (imDCs) and mature DCs (VAX-DC/MM) were assessed by flow cytometry. VAX-DC/MM showed the typical features of mature DCs in morphology with long cytoplasmic projections, eccentric multilobulate lateral nuclei, and abundant cytoplasm compared to imDCs, as previously described (data not shown) [8]. The phenotypic analysis showed that the expression of CD80, CD83, and CD86 were significantly increased in the mature DCs compared to the imDCs. Analysis of lineage markers showed that contamination with B cells (CD19), T cells (CD3) and monocytes (CD14) was < 5% (Figure 2A). The expression of CD80 (94.1%±0.04), CD86 (95.5%±0.03) and CD11c (88.0%±0.04) in 12 individual VAX-DC/MM was high enough to satisfy the identity of DC vaccines according to the quality control of the Vaxcell-Bio Therapeutics (Figure 2B). T cell proliferation capacities of VAX-DC/MM were evaluated by co-culture of CFSE-labeled allogeneic CD3^+^ T cells with VAX-DC/MM at a ratio of 1:4 (DCs: CD3^+^ T cells) for 5 days. The allogeneic CD3^+^ T cell proliferation was efficiently occurred in VAX-DC/MM compared to T cell alone as shown in the representative (Figure 2C; #6 patient - 63.7%) and 12 individual data of VAX-DC/MM (Figure 2D; 72.2%±0.15). To assess the functional potency of VAX-DC/MM, we examined Th1 cytokine and chemokine production, migration capacity, naïve T cell polarization capacity and antigen-specific immune responses by VAX-DC/MM compared to αDC1s, which are well-established type 1-polarized DCs [9]. VAX-DC/MM produced higher levels of IL-12p70 (*, *p<0.05*) and CXCL-10 compared to αDC1s after stimulation with CD40L-transfected J558 cells (Figure 3A). In addition, we performed *in vitro* migration assays using CCR7 ligands, such as CCL21 and CCL19. The VAX-DC/MM showed higher migration ability than αDC1s, in response to CCL21 and CCL19 chemokines (Figure 3B). Naive CD4^+^ T cell differentiation by VAX-DC/MM was evaluated by intracellular staining of IFN-α for Th1 and IL-4 for Th2 polarization, respectively. VAX-DC/MM efficiently skewed naïve CD4 T cells toward IFN-α-secreting Th1 phenotypes comparable to αDC1s (Figure 3C). In the ELISPOT assay to investigate the myeloma-specific immune responses, the number of IFN-γ-secreting cells in CTLs generated by VAX-DC/MM was higher than αDC1s at various E: T ratios (12.5:1, 6.25:1, and 3.125:1) (Figure 3D).

**Figure 1:**
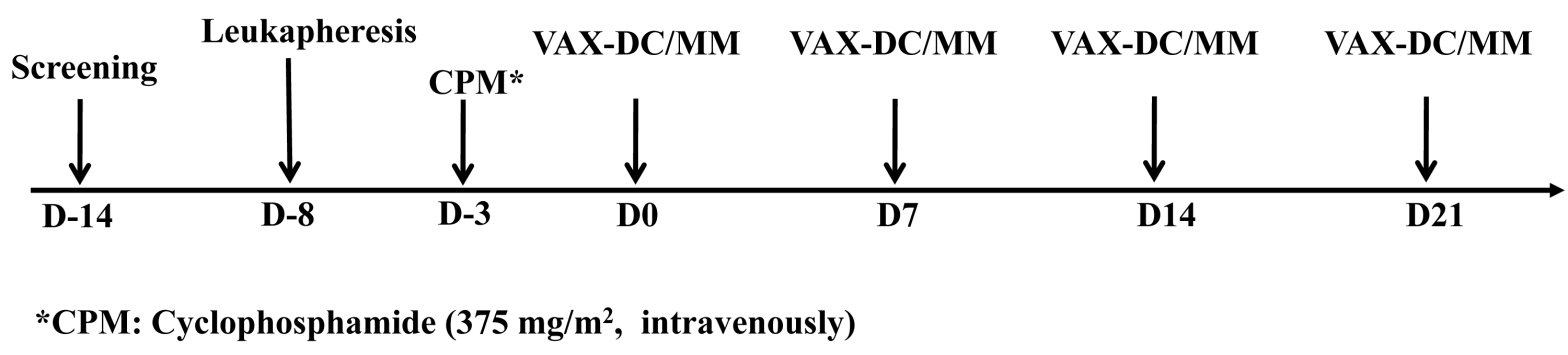
Treatment protocol (Schedule of vaccination)

**Figure 2:**
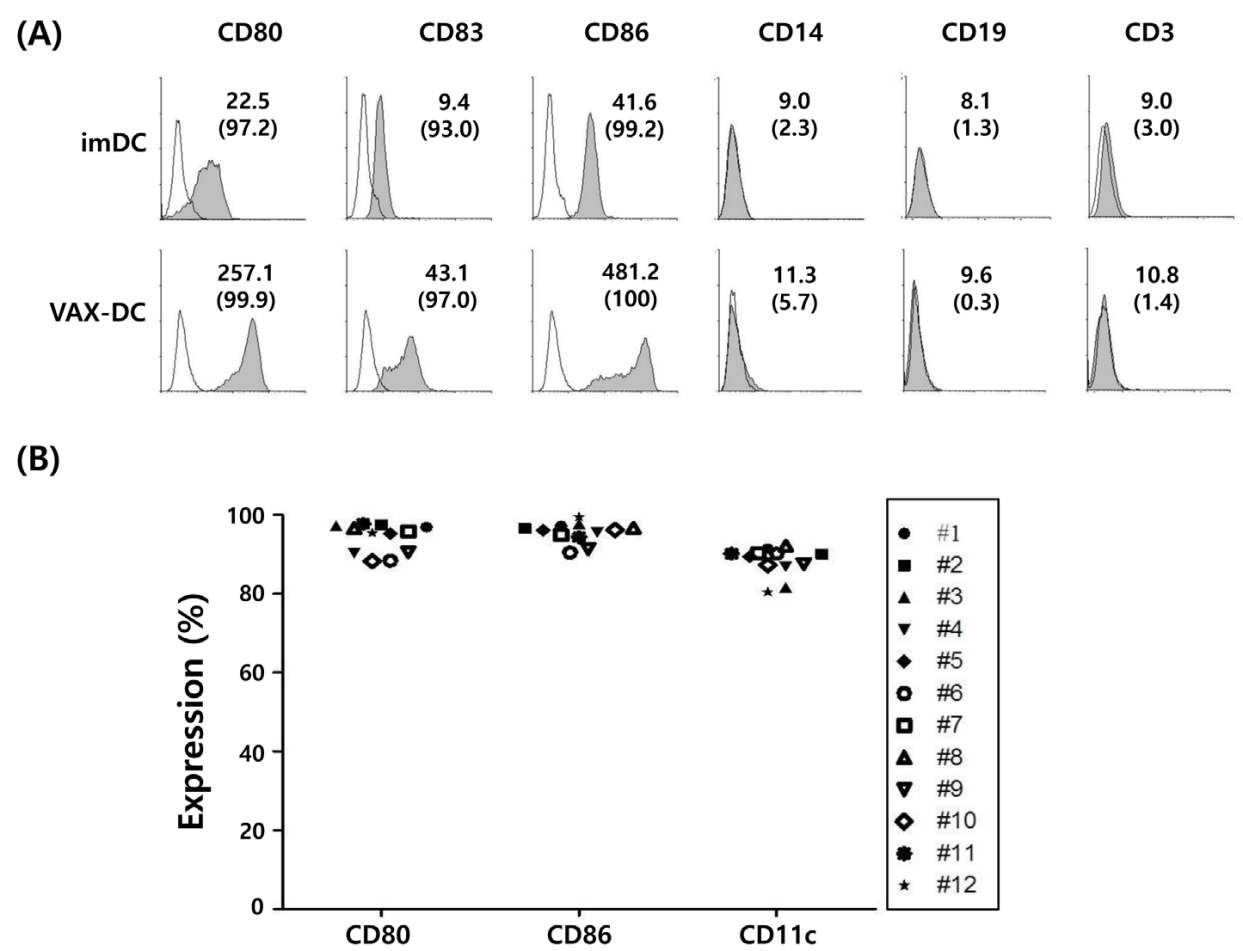

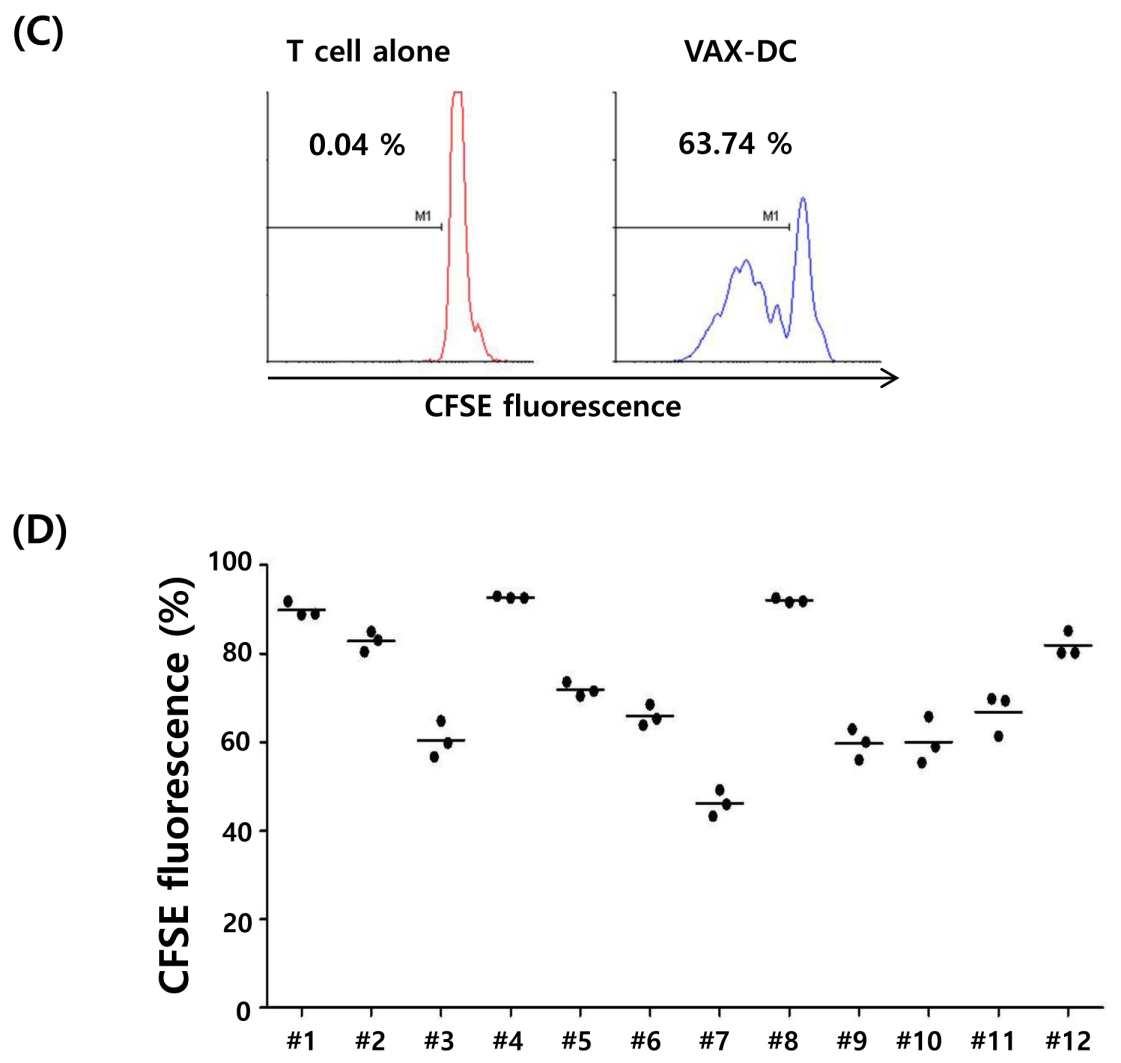
Surface immunophenotypes and T cell proliferation capacities of VAX-DC/MM were shown in the representative and 12 individual data of VAX-DC/MM **A**. and **B**. Expression of surface markers in imDC and VAX-DC/MM was determined by flow cytometry. The value of MFI (upper) and % expression (lower parentheses) was shown. **C**. and **D**. T-cell proliferation capacity was assessed by allogeneic CD3^+^ T cells labeled with CFSE and stimulated with DCs for 5 days at a ratio of 1:4 (DCs: CD3^+^ T cells).

**Figure 3:**
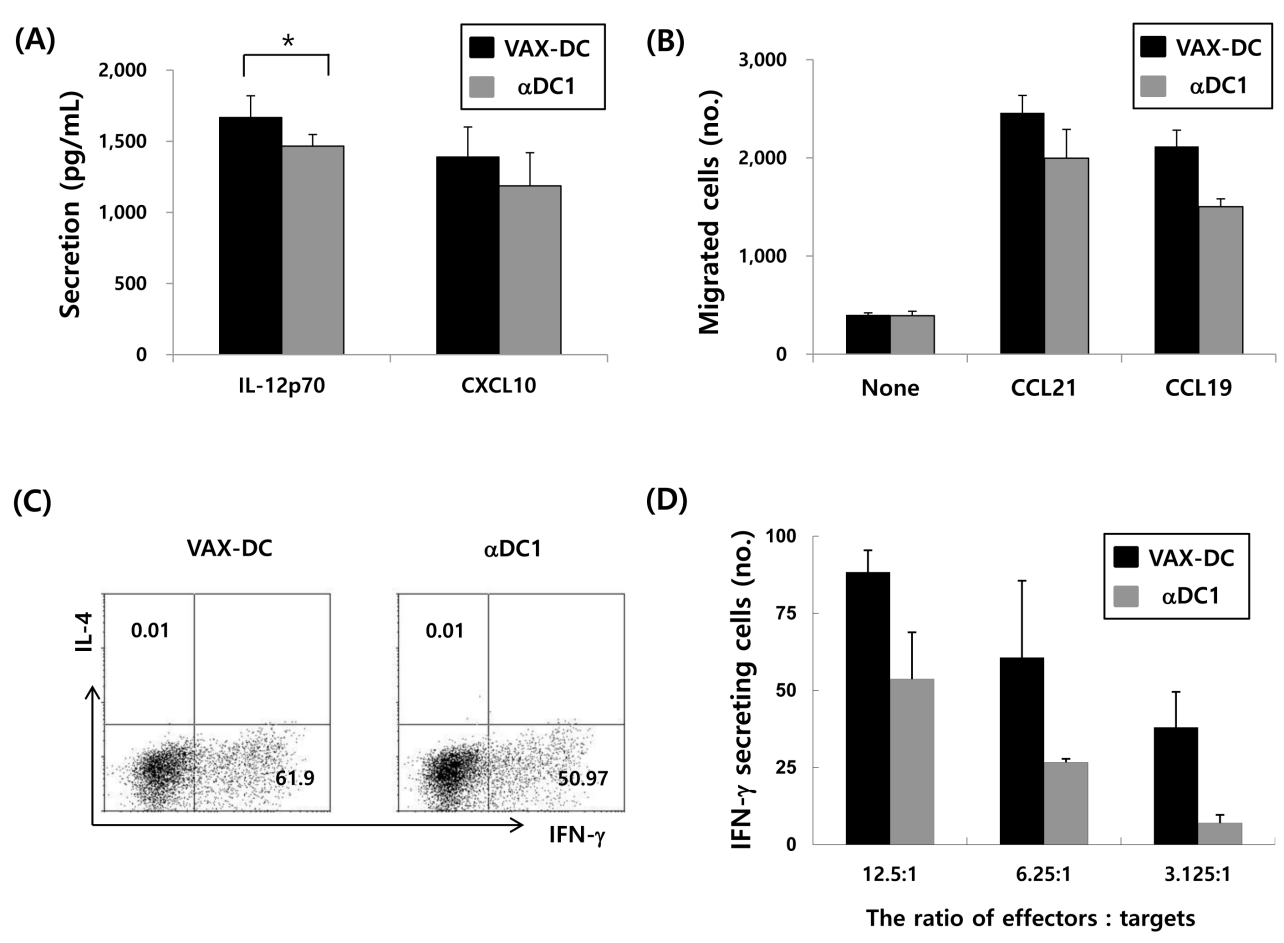
Functional characteristics of VAX-DC/MM **A**. VAX-DC/MM produced higher levels of IL-12p70 (*, *p<0.05*) and CXCL-10 after stimulation with CD40L-transfected J558 cells, compared to αDC1s. **B**. Efficient migration of VAX-DC/MM in response to chemokine CCL21 and CCL19 comparable to αDC1 evaluated by *in vitro* transwell system. **C**. Naïve CD4 T cell polarization by VAX-DC/MM and αDC1 was examined by intracellular staining of IFN-α for Th1 and IL-4 for Th2 after co-culture of allogeneic naïve CD4^+^ T cells for 12 days in the presence of rhIL-2 (10 U/mL). **D**. Myeloma-specific cytotoxic T lymphocytes were evaluated by IFN-γ ELISTOT assay. Data are shown from a representative of three independent experiments.

### Adverse events

Treatment was generally well-tolerated regardless of cell dose of VAX-DC/MM, and there were no grade 3 or 4 adverse events. Hematological and non-hematological adverse events during VAX-DC/MM therapy are summarized in Table 2. The most frequent adverse events were injection-site reactions (12 patients); all were self-limiting and resolved within 1 week. Other common adverse events were myalgia (4 patients), fever (2 patients), and chills (2 patients). Transient grade 1 lymphocytopenia and thrombocytopenia developed in two patients each. Two patients had subclinical hypothyroidism prior to treatment, but VAX-DC/MM therapy did not affect the level of thyroid hormone.

**Table 2:** Treatment-related adverse events (*n* = 12)

	Grade	Number of patients (%)
Injection-site reaction (erythema, itching)	1-2	12 (100%)
Myalgia	1 2	3 (25%) 1 (8.3%)
Fever	1	2 (16.6%)
Chills	1	2 (16.6%)
Pruritus	1	1 (8.3%)
Neutropenia	1	1 (8.3%)
Lymphocytopenia	1	2 (16.6%)
Thrombocytopenia	1	2 (16.6%)

### Immunological and clinical responses

**Figure 4:**
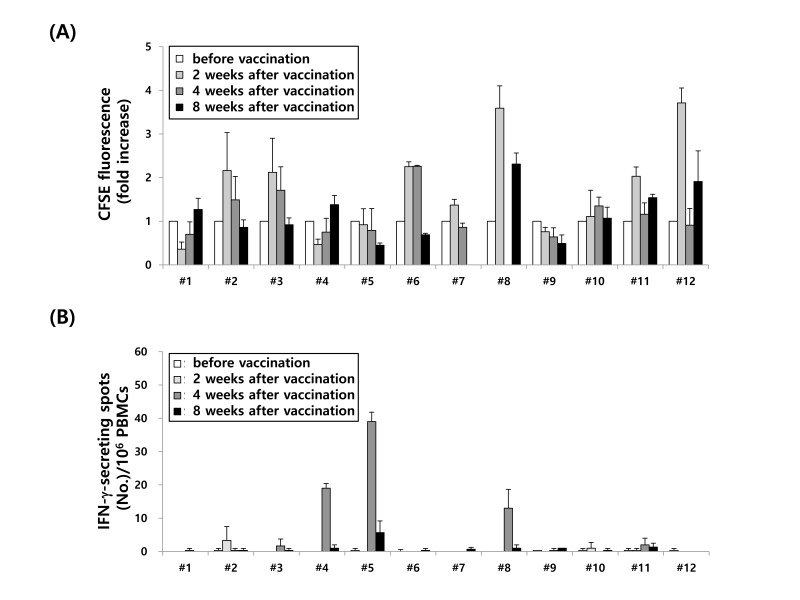
Immunological analysis in 12 MM patients before and after (VAX-DC/MM vaccination **A**. T cell proliferation and **B**. MM-specific immune responses at various ratios of effectors: targets (12.5:1, 6.25:1 and 3.125:1) were evaluated before and after vaccination (2, 4 and 8 weeks) of VAX-DC/MM by CFSE-based MLR at a ratio of 1:4 (DCs: CD3^+^ T cells) for 5 days and IFN-α ELISPOT assays, respectively

Immunological and clinical responses were evaluated in nine patients who received 10 × 10^6^ cells. The results are presented in Table 3. The efficacy and potency of 12 individual VAX-DC/MM vaccines was stable and reproducible before and after vaccination as shown in Figure 4. An immunological response, measured by T-cell proliferation assay or ELISPOT assay, was observed in seven (77.8%) patients after the first VAX-DC/MM injection. Three patients showed immunological responses by the ELISPOT assay and four were detected by the T-cell proliferation assay. The clinical response was evaluated in nine patients treated with 10 × 10^6^ cells. The clinical benefit rate was 66.7%, including one (11.1%) minor response and five (55.6%) with stable disease. In three (33.3%) patients, disease progression was seen. Over a follow-up period of a median 16.1 months (range, 5.5-23.1), eight patients progressed and all patients were still alive. The median progression-free survival was 2.9 months (95% CI = 2.7-3.2 months). Five of eight patients who had progressive disease received salvage therapy. Four patients received lenalidomide and dexamethasone and all had partial responses. One patient received bortezomib retreatment and showed stable disease. Three patients who initially had progressive disease after VAX-DC/MM therapy showed stable disease without further increases in monoclonal paraprotein. These three patients did not receive salvage therapy after VAX-DC/MM therapy and were observed at 18.0, 16.3, and 6.6 months. No significant correlation was observed between immunological results and clinical responses.

**Table 3:** Summarized results of immunological and clinical evaluation

	*Myeloma-specific immunity	#T-cell Proliferation	Best clinical response	Progression	Subsequent therapy	Current status
Patient 4	positive	negative	SD	yes	Rd	alive
Patient 5	positive	negative	SD	yes	Rd	alive
Patient 6	negative	positive	SD	yes	no	alive
Patient 7	negative	positive	SD	yes	no	alive
Patient 8	positive	positive	PD	yes	VCD	alive
Patient 9	negative	negative	PD	yes	Rd	alive
Patient 10	negative	negative	PD	yes	Rd	alive
Patient 11	negative	positive	MR	no	no	alive
Patient 12	negative	positive	SD	yes	no	alive

Abbreviations: SD, stable disease; PD, progressive disease; MR, minor response; Rd, lenalidomide and dexamethasone; VCD, bortezomib, cyclophosphamide, and dexamethasone.

*Myeloma-specific immunity and #*T-cell proliferation were monitored by IFN-*α *ELISPOT assay and CFSE-based MLR assay, respectively. Detailed experimental procedures were described in the section of methods*

## DISCUSSION

DCs are the important regulators of the immune system. They present antigens on their surface with appropriate co-stimulation molecules and initiate cellular immune response through the stimulation of naïve T-cell. Because of their ability to stimulate T-cells, DCs act as links between innate and adaptive immunity [10, 11]. DCs also have an important role in the maintenance of B-cell function and recall response [12, 13]. Thus, DCs are considered an essential target in generating therapeutic immunity against various cancers including MM, and the main focus of this investigation was to develop potent DCs. Potent αDC1s, induced by cytokine cocktails containing IL-1β, TNF-α, INF-α, IFN-γ, and poly I:C, generate strong functional cytotoxic T lymphocytes (CTLs) in several type of cancers and are, on average, 20-fold more active than conventional DCs [14, 15]. In previous studies, αDC1s showed higher expression of several costimulatory molecules or higher production of IL-12p70, improving the induction of tumor-specific and functional CTLs, compared to conventional DCs *in vitro* and *in vivo* [16–18]. However, they have also shown a lower migration capacity than conventional DCs [19], and the use of maturation cytokine cocktails to induce αDC1s is costly. Thus, attempts to improve the characteristic of αDC1s, such as DC migration capacity, IL-12p70 production, and Th1 polarization, are needed to generate a potent DC vaccine. In addition, a reduction in the number of cytokines is needed to reduce the cost of cytokine maturation cocktails. We previously reported that combinations of TLR agonists and IFNs (IFN-α, IFN-γ) synergistically upregulated the expression of CD38 and CCR7, down-regulate CD74 expression, and induce the highest secretion of IL-12p70 [20]. Based on these results, we generated potent DCs (VAX-DC/MM) with high production of IL-12p70 and good migration capacity.

In the presenting study, common toxicity of immunotherapy using VAX-DC/MM were local reactions at the injection site and infusion-related reaction. Vaccination, regardless of the cell dose, was well tolerated without significant toxicity or evidence of autoimmunity. Some patients developed hematological toxicities that were thought to be related to injection of cyclophosphamide before VAX-DC/MM therapy. This safety profile suggests that immunotherapy will become another optional modality because most of patients with relapsed or refractory MM have various complications or frailty due to previous chemotherapy.

Most patients (77.8%) who received 10 × 10^6^ cells showed an immunological response. However, an immunological response did not lead to remarkable improvement in clinical responses. Although clinical benefits were observed in 66.7% of patients including one minor response and five with stable disease (55.6%), most of patients showed disease progression within a short follow-up period. This unsatisfactory clinical outcome may be related to the characteristics of the patients who participated in this clinical trial. Enrolled patients had already had heavily treated relapsed or refractory MM, and had received a median of five prior treatment regimens. Generally, patients with relapse or refractory MM had high tumor burdens, and various immune evasion mechanisms, such as intrinsic alternations and the establishment of an immunosuppressive milieu, thereby limiting the anti-myeloma effect of immune effector cells [21, 22]. Thus, the post-transplantation period may be suitable for the application of immunotherapy because of a lower tumor burden. Recent clinical studies have reported that DC-based vaccines in the post-transplantation period result in a reduction of minimal residual disease or disease stabilization [23, 24]. However, after high-dose chemotherapy and autologous stem cell transplantation, the immune cells are depleted and impaired immune function may last for years [25]. The optimal timing for maximizing the effectiveness of immunotherapy remains unclear.

New treatment strategies are required to enhance response of DCs-based vaccine and to overcome immunosuppressive microenvironment in patients with MM. In this clinical study, all patients treated with lenalidomide-based salvage treatment after VAX-DC/MM therapy showed partial response. Although there were few patients, this response was higher than the response rate reported for lenalidomide-based salvage therapy in heavily pretreated patients with MM [26, 27]. Lenalidomide enhances T cell expansion with Th1 polarization by inhibition of Treg development and PD-1 expression, by activation of NK cells and T-cells, and by suppression of inhibitory factors, and enhances tumor-specific immune responses [28, 29]. In addition, it improves the quality of antigen-specific T cells induced or expanded by functional DCs [30]. These immunological properties of lenalidomide may have affected the response after DC-based therapy. Recently, we reported that a combination of lenalidomide and DC vaccine synergistically improved anti-tumor immunity in a preclinical *in vivo* mouse myeloma model by inhibiting immunosuppressor cells and recovering effector cells [31]. Recent data also suggest that inhibitory receptor blockade by PD-1 inhibition stimulated the proliferation and cytokine secretion of exhausted CD8^+^ T-cells *in vitro* [32, 33]. These results suggest that combination treatment with agents that can modulate the tumor microenvironment could be more effective than monotherapy with a DC-based vaccine in the future.

In this clinical trial, we used DCs loaded with dying MM cells from patients. Whole tumor antigen-loaded DCs have the advantage of allowing the presentation of multiple epitopes to MHC on DCs, thus inducing polyclonal T-cell responses from many potentially unknown tumor-associated antigens [34]. However, it is difficult to obtain an adequate amount of myeloma cells from bone marrow of previously heavily treated patients, and this limitation applies to the treatment of more patients. To overcome these limitations, we are trying to develop recombinant tumor-associated antigens (TAAs), which are abundantly expressed in myeloma cells of patients with MM, using efficient delivery tools to increase the immunogenic sensitivity of TAAs for DCs. DCs loaded with these multiple TAAs may be another promising tool for use in immunotherapy.

In conclusion, VAX-DC/MM therapy, regardless of cell dose, was well-tolerated, and most patients showed an immunological response and disease stabilization. Further studies are needed to increase the efficacy of VAX-DC/MM in patients with MM, possibly in combination with immunomodulatory agents and check-point blockade.

## PATIENTS AND METHODS

### Patients

This study was a prospective, open-labelled phase I clinical trial (ClinicalTrials.gov ID: NCT02248402). All patients provide written informed consent before enrollment, and the study protocol was approved by the Institutional Research Board of Chonnam National University Hwasun Hospital. All procedures associated with this study were conducted in accordance with the principles of the Declaration of Helsinki and local law.

Eligible patients were at least 18 years old and had relapsed or refractory MM, who had received both thalidomide- and bortezomib-containing regimens. Other eligibility criteria included Eastern Cooperative Oncology Group performance status ≤ 2, platelet count ≥ 50 × 10^9^/L, absolute neutrophil count ≥ 1.0 × 10^9^/L, aspartate aminotransferase, alanine aminotransferase, and alkaline phosphatase < 3 times the upper limit of normal, and creatinine clearance ≥ 20 mL/min. We excluded patients with active infectious disease, clinically significant autoimmune disease, pregnant females, and those who declined to participate.

### Study design

Treatment protocol is summarized in Figure 1. To create VAX-DC/MM, myeloma cells were obtained from the bone marrow of the participants within 2 weeks before the first injection of VAX-DC/MM. Peripheral blood mononuclear cells from the patients were collected by leukapheresis at D-8. Before the first injection of VAX-DC/MM, cyclophosphamide (375 mg/m^2^, intravenously) was administered to modulate immune response at D-3. Patients received the intradermal VAX-DC/MM injection every week for 4 weeks. Three patients each were treated with 5 × 10^6^, and 10 × 10^6^ cell. After the higher dose was established as the tolerable dose, an additional six patients were enrolled and received 10 × 10^6^ cell doses.

### VAX-DC/MM generation

After performing leukapheresis to the patients, peripheral blood mononuclear cells (PBMC) were isolated by density gradient centrifugation at 1200×g for 25 min on lymphoprep™ (Axis-Shield, Oslo, Norway) solution. Monocytes were separated from PBMCs by density gradient centrifugation using percoll^®^ (GE healthcare Bioscience AB, Uppsala, Sweden) solution and were allowed to adhere to culture flasks. After 30 min, non-adherent cells were removed and adherent cells were culture to generated DCs in serum-free medium, CellGenix™ GMP DC (Cellgenix, Freburg, Germany) with 50 ng/mL recombinant (rh) GM-CSF (JW CreaGene Inc, Gyeonggi-do, Republic of Korea) and 25 ng/mL rhIL-4 (JW CreaGene Inc). On day 5, CD138^+^ cells were isolated from bone marrow mononuclear cells of patient with MM using CD138 microbeads (Militenyi Biotec, Bergisch Gladbach, Germany), as a tumor antigen, irradiated with UVB (120 mJ/cm^2^), and then incubated overnight at 37°C in humidified 5% CO_2_ incubator as described previously [19, 34]. On day 6, the immature DCs were harvested and activated by addition of LPS at 1 μg/mL (Sigma Aldrich, St. Louis, MO, USA), polyinosinic:polycytidylic acid [Poly(I:C)] at 20 μg/mL (Sigma Aldrich), Roferon^®^-A (recombinant IFNα-2a) at 3,000 U/mL (Roche, Wurmisweg, Switzerland), and IFN-γ at 200 U/mL (Peprotech, Rocky hill, USA), and were loaded with UVB-irradiated dying myeloma cells and keyhole limpet hemocyanin (KLH, Sigma Aldrich) protein (5 ng/mL) after 2 h. On day 8, mature DCs were harvested and cryopreserved in liquid nitrogen until used. For the generation of alpha-type 1-polarized DCs (αDC1s) [9], IL-1β at 25 ng/mL (Peprotech), TNF-α at 50 ng/mL (Peprotech), Roferon^®^-A at 3000 U/mL (Roche), IFN-γ at 1,000 U/mL, and Poly(I:C) at 20 μg/mL were used on day 6. The percentage and activation-related markers of DCs, including FITC-conjugated anti-CD14, FITC- conjugated anti-CD3, FITC-conjugated anti-19, FITC-conjugated anti-CD11c, PE-conjugated anti-CD80, PE-conjugated anti-CD83, and PE-conjugated anti-CD86 (all antibodies obtained from BD pharmingen, San Jose, CA, USA) were analyzed using a FACSCalibur flow cytometer (BD, New Jersey, USA) with WinMDI software (Biology Software Net).

### Quality control of VAX-DC/MM

All generated DCs underwent safety tests including endotoxin, mycoplasma, and sterility test, and tests for adventitious agents. Sterility, phenotypic analysis for identity and purity, cell viability profiles of VAX-DC/MM were evaluated according to the standard operating protocols and test guidelines of Research Institute, Vaxcell-Bio Therapeutics, approved by the Korea Ministry of Food and Drug Safety.

### Cytokine and chemokine production from VAX-DC/MM

VAX-DC/MM and αDC1s were cultured in 96-wells plates at 2 × 10^4^ cells/well and stimulated with CD40 ligand (CD40L)-transfected J558 cells (as an analog of CD40L-expressing Th cells; a gift from Dr. P. Lane, University of Birmingham, UK) at 5 × 10^4^ cells/well [9]. After 24h, supernatants were collected and levels of IL-12p70 (BD Biosciences, San Jose, CA, USA) and CXCL-10 (R&D Systems, Minneapolis, MN) were determined by enzyme-linked immunosorbent (ELISA) assay. Each sample was analyzed in triplicate and the mean absorbance for each set of standards and samples was calculated.

### Migration assay of VAX-DC/MM

Chemokine-induced migration of VAX-DC/MM and αDC1s was performed using 24-well transwell plates with polycarbonate inserts of 5-μm pore size (Corning Costar, Cambridge, MA, USA). A total of 600 μL of culture medium (IMDM with 10 % FBS) with CCL21 (250 ng/mL, Peprotech) or CCL19 (250 ng/mL, Peprotech) was added to the bottom of the chambers. Chemokine-free culture medium served as a control. The DCs (5 × 10^4^ cells/100 μL) were added to the upper chamber. After incubation at 37°C for 3 h, the migrated DCs (500 μL) in the bottom chamber were collected and counted using the FACSCalibur for 60 s.

### Induction of myeloma-specific CTLs by VAX-DC/MM

CD8^+^ T cells (purity > 96%) were negatively isolated using MACS (Miltenyi Biotec) from isolated PBMC. CD8^+^ T cells (5 × 10^5^ cells/well) were sensitized with autologous VAX-DC/MM and αDC1s (5 × 10^4^ cells/well) loaded with dying U266 cells. After 3 days, rhIL-2 and rhIL-7 (Peprotech) were added to the wells of the 24-well culture plate. On day 10, cultured CD8^+^ T cells were restimulated with the same DCs. On day 20, myeloma-specific CTLs for VAX-DC/MM and αDC1s were analyzed using U266 cells as targets cells by IFN-γ enzyme-linked immunospot (ELISPOT) assay (BD Biosciences). The spots were counted using an automatic CTL Immunospot Reader (CTL-ImmunoSpot® S6 Core Analyzer, Cellular Technology LTD, Shaker Heights, OH, USA). The ELISOPT data are expressed as the mean (±SD) number of spots per 1 × 10^5^ T cells.

### Immunologic monitoring after vaccination

An ELISPOT assay of IFN-γ and T-cell proliferation was performed using PBMCs collected from patients prior to vaccination and at 2 weeks, 4 weeks, and 8 weeks after the first VAX-DC/MM injection. The mature DCs and PBMCs were cryopreserved until use.

For the ELISPOT assay, the 96-well plate of the ELISPOT kit (BD pharmingen, San Jose, CA, USA) was coated with 200 μL/well anti-IFN-γ antibody overnight at 4°C under light protection, washed with PBS containing 0.1% Tween-20 (Amresco, solon, Ohio) and blocked with 200 μL RPMI 1640 medium supplemented with 10% FBS, 1% penicillin/streptomycin, at 37°C in a humidified 5% CO_2_ incubator for 2 h. The PBMCs, as responder cells, were stimulated with DCs and KLH in a 96 -well culture plate coated with IFN-γ antibody overnight at 37°C in a humidified 5% CO_2_ incubator. Definitely positive spots stained with an anti-IFN-γ antibody were analyzed with an automatic CTL ImmunoSpot Reader (CTL-ImmunoSpot® S6 Core Analyzer, Cellular Technology LTD).

For the T-cell proliferation assay, the PBMCs of patients were labeled with carboxyfluorescein succinimidyl ester (CFSE, Oregeon, invitrogen) according to the manufacturer's protocol. Then, labeled PBMCs were cultured at 2 × 10^5^ cells/well in 100 μL RPMI 1640 medium supplement with 10% FBS and 1% penicillin/streptomycin in a 96-well plate and activated with mature DCs (5 × 10^4^ cells/well) at 37°C in humidified 5% CO_2_ incubator for 5 days. After 5 days, cultured cells were analyzed using the FACSCalibur (BD) and the WinMDI software (Biology Software Net).

### Assessment of clinical response after VAX-DC/MM vaccination

International Myeloma Working Group response criteria were used to assess the treatment response [35]. Disease status was initially assessed at D-14, and then every 4 weeks from first injection of VAX-DC/MM until the time of progression. Toxicity was assessed and graded according to the National Cancer Institute Common Toxicity Criteria (NCI-CTC v4.0) from the first injection of VAX-DC/MM to the end of the study.

### Statistics

The Mann-Whitney *U*-test was used to calculate the statistical significance of non-parametric differences. All statistical analyses were performed using the SPSS software (ver. 13.0; SPSS, Inc., Chicago, IL, USA). A *P*-value < 0.05 was considered to indicate statistical significance in all analyses.

## SUPPLEMENTARY MATERIALS


