# Supplementary material for: A phase I clinical study of autologous dendritic cell therapy in patients with relapsed or refractory multiple myeloma
Source: Oncotarget. 2017 Jan 10;8(25):41538–48. doi: 10.18632/oncotarget.14582 (PMC5522196; doi:10.18632/oncotarget.14582)
Supplement: Supplementary file 1 [file oncotarget-08-41538-s001.pdf]

A phase I clinical study of autologous dendritic cell therapy in patients with relapsed or refractory multiple myeloma

Quality control of VAX-DC/MM:

Safety testing of 12 VAX-DC/MM for viability, sterility (mycoplasma, endotoxin, virus, bacteria), potency (T cell proliferation), identity (surface phenotypes) and purity (lineage markers) was evaluated according to the standard operating protocols and test guidelines of Research Institute, Vaxcell-Bio Therapeutics and Jeonnam Biopharmaceutical Research Center (JBRC, Hwasun, Jeollanamdo, Republic of Korea), approved by the Korea Ministry of Food and Drug Safety.

Sterility test of VAX-DC/MM

Membrane filtration method was performed by Membrane Filtration Sterility kit (Merck Millipore, USA) after 14 day culture for the environmental monitoring of VAX-DC/MM.

Agar and semi-solid broth methods (Sigma-Aldrich, Canada) were applied to detect mycoplasma contamination after 28 day culture. M. pneumoniae and M. orale at ≤ 100 CFU were used as positive controls.

Virus contamination was examined by Hemagglutination assay using chicken and guinea pig red blood cells and Cell culture test (observation of morphological changes) using vero (a monkey kidney epithelial cell line) and MRC-5 (a human diploid cell line), respectively.

Gram stain kit (BD pharmingen, CA, USA) was employed for the detection of bacterial contamination.

Endotoxin test was determined by Limulus Amoebocyte Lysate (LAL) endotoxin assay kit (Endosafe; Charles River Laboratories, Wilmington, MA). The amount of endotoxin was formulated with endotoxin units per ml (EU/ml).

Cell viability of VAX-DC/MM

Cell viability of VAX-DC/MM was evaluated by trypan blue exclusion method under the microscope.

Identity and purity of VAX-DC/MM

The percentage and mean fluorescence intensity (MFI) of VAX-DC/MM for identity and purity were analyzed by a FACSCalibur flow cytometer (BD Biosciences, New Jersey, USA) with WinMDI software (Biology Software Net). FITC-conjugated anti-CD14 (monocytes), anti-CD3 (T lymphocytes), and anti-CD19 (B lymphocytes) were used as lineage makers for purity of VAX-DC/MM. PE-conjugated anti-CD11c, anti-CD80, and anti-CD86 were used as DC activation and maturation markers for identity of VAX-DC/MM (all antibodies obtained from BD pharmingen, San Jose, CA, USA). Isotype-matched controls (mouse IgG1 and mouse IgG2, BD) were used in parallel.

Potency of VAX-DC/MM

Mixed lymphocyte reaction (MLR) of VAX-DC/MM was performed for the potency of VAX-DC/MM using co-culture of CFSE-labeled allogeneic CD3+ T cells with VAX-DC/MM at a ratio of 1:4 (DCs : CD3+ T cells) for 5 days and was analyzed by a FACSCalibur flow cytometer (BD Biosciences, New Jersey, USA)
